# From neighborhood to household: connections between neighborhood vacant and abandoned property and family violence

**DOI:** 10.21203/rs.3.rs-4022003/v1

**Published:** 2024-03-11

**Authors:** Julia M Fleckman, Julie Ford, Sophia Eisenberg, Catherine A. Taylor, Michelle Kondo, Christopher N. Morrison, Charles C. Branas, Stacy S. Drury, Katherine P. Theall

**Affiliations:** Tulane University School of Public Health and Tropical Medicine; Tulane University School of Social Work; Boston College School of Social Work; Boston College School of Social Work; Northern Research Station USDA Forest Service: USDA Forest Service Northern Research Station; Columbia University Mailman School of Public Health; Columbia University Mailman School of Public Health; Boston Childrens Hospital: Boston Children’s Hospital; Tulane University School of Public Health and Tropical Medicine

**Keywords:** Family violence, intimate partner violence, child maltreatment, vacant property, neighbourhood disorder

## Abstract

Rates of family violence, including intimate partner violence (IPV) and child maltreatment, remain high in the U.S. and contribute to substantial health and economic costs. How neighborhood environment may influence family violence remains poorly understood. We examine the association between neighborhood vacant and abandoned properties and family violence, and the role collective efficacy may play in that relationship. Data were used from a longitudinal cohort of 218 maternal-child dyads in a southern U.S. city known for elevated rates of violence. Women were matched on their propensity score, for living in a neighborhood with elevated vacant and cited properties. Analyses accounting for clustering in neighborhood and matched groups were conducted to examine the association between neighborhood vacant and abandoned property and family violence, and the potential mediating relationship of collective efficacy. The likelihood of experiencing child maltreatment at 12-months of age was more than twice as high for children living in neighborhoods with a high vacant and cited property rates compared to women living in neighborhoods with fewer vacant and cited properties (OR=2.11, 95% CI=1.03, 4.31). Women living in neighborhoods characterized by high levels of vacant and cited properties were also more than twice as likely to report IPV (OR=2.52, 95% CI=1.21, 5.25). Associations remained mostly stable after controlling for key covariates. Collective efficacy did not act as a mediator in the relationship between vacant and cited properties and family violence. Reducing neighborhood vacant and cited properties may be an important target for interventions focused on reducing family violence.

## Introduction

Family violence—which includes intimate partner violence (IPV) and child maltreatment—is a global public health crisis contributing to increasing trends in violence and associated health and economic costs to society. In the United States, nearly half of women and more than 40% of men have experienced some form of IPV in their lifetime, including contact sexual violence, physical violence and/or stalking.^1^ For women, lifetime prevalence of physical violence by an intimate partner was an estimated 42%. Nearly one third of women reported experiencing at least one act of severe physical violence by an intimate partner during their lifetimes. Additionally, nearly half of the women (49.4%) and men (45.1%) surveyed reported experiencing psychological aggression—the most common form of IPV—during their lifetimes.^1^ Further, child maltreatment exacts enormous human and economic costs, threatening physical, mental, and behavioral health, and contributing to epigenetic changes and altered brain and neurophysiological development in children.^2^ According to the Children’s Bureau’s^3^ summary of 2022 National Child Abuse and Neglect Data System (NCANDS) data, there were over half a million victims of child abuse and neglect, equivalent to a rate of 7.7 victims per 1,000 children nationally.

A growing body of literature has demonstrated the influence of neighborhood context on neighborhood socioeconomic inequities, norms that promote violence and aggression, and various forms of violence. Findings that point to high levels of crime consistently remaining in neighborhoods with high poverty, few institutional supports, and weak community ties have led the shift to more critically examining neighborhood context,^4^ with an examination of the structural role of long-term disinvestment in creating areas of concentrated disadvantage especially in urban areas.^5^ Neighborhood physical disorder, including vacant or visibly damaged housing or commercial buildings, litter or trash, and graffiti,^6^ can be considered a downstream effect and marker of this structural disinvestment and concentrated disadvantage.^7^ Physical and environmental have been directly linked to forms of violence that occur behind closed doors, such as intimate partner violence and child maltreatment.^8,9^ Markers of neighborhood socioeconomic status have been strongly linked to both IPV and child maltreatment, including neighborhood deprivation, poverty, and unemployment status.^10^ Studies examining the impact of disordered neighborhoods have also found higher rates of child maltreatment in areas with more neighborhood disorder.^11,12^

Collective efficacy, thought to influence levels of informal social control within neighborhoods and account for neighborhood-level variations in disorder,^13^ is understood as the presence of shared values and mutual trust among neighbors alongside a general willingness to intervene when socially unacceptable or violent behavior is witnessed.^6,13^ An association has been found between higher levels of collective efficacy and lower levels of street crime, and collective efficacy can mediate the effects of concentrated disadvantage and residential instability on violence.^13^ Conversely, the relationship between collective efficacy and family violence has been less explored, with extant research yielding mixed resultse.^e.g.,8,14,15^ Despite some research demonstrating that neighborhood collective efficacy can act as a protective factor against IPV,^8^ additional research suggests collective efficacy does not appear to act as a strong mediator between neighborhood structural factors and IPV.^15^ There may be important differences in how neighborhood social ties influence neighborhood violence versus IPV, where some forms of IPV might not be considered by neighbors as a community-level safety threat compared with other types of neighborhood violence.^15^ Results have also been mixed regarding the relationship between neighborhood factors, collective efficacy, and the risks of child maltreatment.^14,16^ In particular, Emery et al.^14^ questioned whether the well-documented relationship between collective efficacy and street crime can so easily be translated, suggesting, community members may respond differently to public social problems versus matters considered private or within the family.

Yet, even within this relatively limited field of research, some common themes have emerged. Namely, levels of family violence, including IPV and child maltreatment, appear to be higher in neighborhoods facing concentrated disadvantage, long-term disinvestment, high levels of violent crime, and general signs of social disorder. Given that physical disorder is typically associated with concentrated disadvantage and higher levels of violence, identifying associations between levels of family violence and neighborhood physical disorder can add to the dearth of study in this emerging area. Further, the role of neighborhood processes, such as collective efficacy, in the relationship between neighborhood disorder and family violence has not been well explored and has ceded mixed results. The current study adds to the literature by examining the associations between neighborhood vacant and abandoned properties and family violence in a longitudinal cohort of maternal-child dyads in a southern U.S. city known for elevated rates of violence. Our hypothesis is that women living in neighborhoods with high rates of vacant and cited property will have an increased risk of family violence. Additionally, we examine parental stress and collective efficacy as potential mediators in the relationship between neighborhood vacant and cited properties and family violence. A deeper understanding of these relationships may inform the design and planning of neighborhood place-based interventions, such as improvements to built or natural environments, infrastructure, or amenities that may help reduce family violence.

## Methods

### Study Design and Sample

The current secondary study was based on a sample of 395 mothers ages 18 to 43 years recruited from prenatal clinics, Women, Infants, and Children (WIC) clinics, and other ongoing University studies between years 2012 and 2017 in New Orleans, Louisiana. The parent study from which this data for this secondary analysis were sourced examined the impact of cumulative maternal stress and attachment on birth outcomes and childhood behavioral and health outcomes (XXXX). Mothers provided information about multiple levels of their and their infant’s social ecology using an interview-assisted computer survey administered face-to-face by a trained research assistant. Surveys were conducted at four time points: during pregnancy, and when the child was 4-, 12- and 18-months of age. This study was approved by the University Institutional Review Board.

The current study further restricted the sample to those participants who provided a complete residential address during pregnancy and who resided in New Orleans (N=218), given that data on vacant and cited property exposure were only available within the city limits. Addresses were geocoded to link respondents to their residence and linked to their census tract or neighborhood. There was an average of 2 respondents per census tract (range=1 to 6).

To minimize selection bias or structural confounding, given the neighborhood exposure of interest, a propensity score analysis^17^ was conducted to examine overlap in the propensity for living in a neighborhood with high vacant and cited property rates. Propensity models included individual and neighborhood socioeconomic indicators such as neighborhood concentrated disadvantage and level of urban life stressors, as well as maternal education, family annual income, age, and race and ethnicity. Mothers were matched 2:1 (with replacement) on the propensity score (with a caliber of 0.05). Accounting for missing data in the outcomes, the final matched sample included 139 observations for examination of child maltreatment and 429 observations for examination of IPV.

### Measures

#### Outcomes

**IPV experiences of the mother** were assessed at baseline (during pregnancy), 4-, and 12-months follow up with the *Hurt, Insult, Threaten, and Scream* (*HITS*) *Domestic Violence Screening Tool*.^18^The HITS screening tool is a validated, reliable four-item instrument that asks, “How often does your partner: physically hurt you, insult you or talk down to you, threaten you with harm, and scream or curse at you?”.^18^ Participants are asked to give responses on a five-point frequency scale: *(1) never, (2) rarely, (3) sometimes, (4) fairly often and (5) frequently*. Answers to all four items are summed, resulting in scores ranging from 4 to 20. For the purposes of the present analysis, the variable was dichotomized so that participants who reported any abuse during pregnancy or after pregnancy were coded as (1) has experienced IPV and all other responses were coded as (0) has not experienced IPV.

**Child maltreatment** was assessed using 20-items from three *Parent-Child Conflict Tactics Scale (CTS)*^19^ sub-scales: psychological aggression, physical assault, and neglect. The measure was administered when the child was 12-months of age to gather how often in the past year the caregiver engaged in the assessed behaviors. Answer choices included: *(0) never, (1) once, (2) twice, (3) 3–5 times 6–10 times, (5) 11–20 times, (6) more than 20 times, and (7) not in the last year*. A positive report of any of the three types of behaviors in the past year (responses 1–7) was coded as 1, otherwise 0 (response 0).

#### Exposure

**Neighborhood vacant and cited property** was measured by the rate of vacant or cited property per 1000 population in the participant’s census tract or neighborhood, calculated based on publicly available data from the City of New Orleans. Rates were calculated and averaged across years of the cohort data collection. Properties included those with a judgment by the City’s Code Enforcement Division for lots that were unoccupied (usually vacant with no structure) and that had one or more of the following violations: grass or vegetation growth higher than 18 inches tall; trash, debris, or evidence of illegal dumping; and/or growth of noxious vegetation, such as poison ivy. The rate also included records of parcels with code violations, with an average of 28 violations per 1000 in a census tract (range=0 to 165). The rate was also dichotomized based on the 75^th^ percentile (38 per 1000 population) to characterize neighborhoods as having high vs. lower vacant and cited property rates.

#### Mediators

**Perceived collective efficacy** was examined as a potential mediator between neighborhood vacant and cited property and family violence outcomes. It was measured at baseline, 4-, and 12-months using 10 items, five aimed at measuring social cohesion and five informal social control.^20^ Social cohesion items were measured on a 4-point scale (*0–3*) from *strongly disagree to strongly agree*, with respondents indicating the degree to which people in the neighborhood share the same values, are willing to help neighbors, can be trusted, and that it is a close-knit neighborhood and gangs are not a problem in the neighborhood. Informal social control items were measured on a 4-point scale (*0–3*) from *very unlikely to very likely* with respondents indicating the likelihood that their neighbors would intervene if youth were skipping school and hanging out in the street, if youth were spray painting graffiti on local buildings, if children were showing disrespect to an adult, if a fight broke out in front of a house, and if the local fire station was threatened with budget cuts. Items were summed to create an index, ranging from 0 to 30.

#### Covariates

Several factors were also examined as potential confounders in the relationship between neighborhood vacant and cited property and family violence outcomes, and all were measured via self-report at baseline. **Sociodemographic factors** included maternal age, education, relationship status, race, ethnicity, and income. Race was based on self-reported, close-ended categorical questions that asked participants if they did or did not identify as a member of specific racial and ethnic groups, with the option of selecting more than one group. Race was first recoded into a categorical variable including all races identified, and given the sample distribution, subsequently recoded into three categories: Black, White, and Other. Marital status was a categorical question that included: *(0) married, (1) married but live separately, (2) single/never married, (3) single/divorced, (4) widow, (5) in a committed relationship/never been married, and (6) in a committed relationship/also divorced or widowed*. Based on responses to the question on marital status, a dichotomous variable was created including (0) single and (1) married/in a relationship.

**Maternal exposure to adverse childhood experiences** was assessed using the *Adverse Childhood Experiences Survey (ACE)*, which was developed by Kaiser Permanente, in conjunction with the Centers for Disease Control and Prevention (CDC), as part of the Adverse Childhood Experiences Study.^21^ Survey items examined a variety of hardships experienced during childhood prior to age 18 including abuse, neglect, and household dysfunction. Of the 26 items in this scale, 10 had binary response options including: *(0) no* and *(1) yes* and sixteen had frequency of occurrence response options with a 5-point Likert scale (*(1) never, (2) once or twice, (3) sometimes, (4) often, and (5) very often*); these responses were also dichotomized (i.e., never = 0). A dichotomous variable was created in which participants who endorsed less than four items were coded as (0) and those that endorsed four or more were coded as (1). Prior studies have suggested an increase in poor health outcomes following report of four or more ACEs.^22^

**Perceived stress** at baseline, 4-, and 12-months was measured with the 4-item *Perceived Stress Scale*.^23^ Respondents were asked how often in the last month they felt: “… that you were unable to control the important things in your life?”, “…confident about your ability to handle your personal problems?”, “… that things were going your way?”, and “… that difficulties were piling up so high that you could not overcome them?”.^23^ Response options ranged from *(0) never to (4) very often*. The second and third items were reverse coded, and a summary score ranging from 0 to 12 was created for all items, with a higher score indicating increased stress.

**Anxiety** was measured with the *Rini Pregnancy-related Anxiety Scale*.^24^ The 10-item scale with reliable internal consistency was designed specifically for use in pregnancy to assess the frequency of, or the extent to which, a woman worries or feels concerned about her health, the health of her baby, labor and delivery, and caring for the baby following delivery. Response options ranged from *(1) never or not at all* to (*4) a lot of the time or very much*. A summary score is created which includes reverse scoring the first and second item. Scores range from 10 – 40, with higher scores suggesting increased anxiety.

### Statistical Analysis

Univariate, bivariate, and multivariate analyses were performed using SAS version 9.4, including generalized estimating equations (GEE) to take into account neighborhood clustering and conditioning on matched stratum. Standard errors, 95% confidence intervals and unless otherwise stated, a p-value <0.05, were used to define statistically significant associations. Logistic regression models analyzed dependent variables IPV and child maltreatment as binary outcomes (1=reported IPV/maltreatment, 0=no IPV/maltreatment). Vacant and cited property was coded both as a continuous measure (Models 1 and 3) and dichotomized into high (75th percentile and above) and low blight areas (Models 2 and 4). Additionally, we examined the mediating role of collective efficacy. Potential mediation by collective efficacy was calculated utilizing the SAS MEDIATE Macro.^25^All covariates were included in final adjusted models.

## Results

Sociodemographic characteristics of the sample are presented in Table 1. The mean age of study participants was 26.8 years (Range: 18 to 43 years). Most of the women identified as Black (65%) and over one-third (40%) had a high school education or less. Half of the sample (50%) had an annual household income of $24,999 or less. Over half (53%) were in a relationship or married. Approximately 18% endorsed four or more ACEs, 46% reported at least one type of child maltreatment, and 36% reported experiencing at least one form of IPV.

Table 2 presents both crude and adjusted results examining the association between neighborhood vacant and cited property rate, and both child maltreatment and IPV. The likelihood of experiencing physical maltreatment, psychological maltreatment, or neglect at 12-months of age was more than twice as high for children living in neighborhoods with a high vacant and cited property rate (OR=2.11, 95% CI=1.03, 4.31, p<0.05), increasing by 1% for each unit increase in vacant and cited property rate; after controlling for key covariates, the adjusted rate was also statistically significant (aOR=9.20, 95% CI=1.03, 82.44, p<0.05). Women living in neighborhoods characterized by high levels of vacant and cited property were also more than twice as likely to report IPV compared to women living in neighborhoods with fewer vacant and cited properties (OR=2.52, 95% CI=1.21, 5.25, p<0.05), increasing by 2% for each unit increase in vacant and cited property rate; after controlling for key covariates, the adjusted rate was attenuated (aOR=1.85, 95% CI=0.88, 3.88, p=0.10).

We examined the mediating role of collective efficacy in both relationships—between vacant and cited property rate and both child maltreatment and IPV—and there was no significant indirect effect for collective efficacy in the relation between vacant and cited property and child maltreatment (indirect effect estimate=1.01, p=0.84) or IPV (indirect effect estimate=1.10, p=0.43).

## Discussion

The results of this study are consistent with the proposed hypothesis that women living in neighborhoods with high rates of vacant and cited property exhibit an increased risk of family violence. Few studies have examined this association even though neighborhood characteristics are important elements of the built environment to examine ^10^ and also have been linked to crime and violence.^26^ Children in neighborhoods with higher levels of vacant and cited property were more than twice as likely to experience physical maltreatment, psychological maltreatment, or neglect at 12-months of age, than those living in neighborhoods with lower levels. Similarly, women living in neighborhoods with high levels of vacant and cited property were two and a half times as likely to respond that they had experienced IPV, compared to women living in neighborhoods with lower levels. Therefore, we can conclude that women living in neighborhoods in our study area with more vacant and cited property experience higher rates of family violence. We also hypothesized that collective efficacy might help to explain the associations between neighborhood conditions and family violence. However, when we tested this potential pathway, we observed no significant indirect effects in this sample, although this may be due to a small sample size.

This study is focused on characteristics of the built environment, so it is important to contextualize our findings within a broader exploration of the root causes of vacant and cited properties, namely, targeted, and chronic disinvestment in community well-being. The built environment is impacted by broader societal factors, such as structural inequity and racism.^27^ Historically, community disinvestment has disproportionately impacted communities with higher populations of people of color.^28^ Additionally, although Black women made up 65% of this study’s overall sample, 83% of them lived in neighborhoods with high rates of vacant and cited property. The overrepresentation of people of color living in physically disordered neighborhoods in this study reflects the influence of structural inequities including redlining, chronic disinvestment, and racial segregation.^28,29^ Moreover, this specific manifestation of structural inequities contributes to social and health disparities, including the risk of exposure to violence, among people of color.^30^ Further, a substantial proportion of participants living in neighborhoods with high vacant and cited property had less than a high school education (35%) or an annual household income of $24,999 or less, both of which are also social determinants of health outcomes.^31^

While this study provides important and novel insights into the connection between the neighborhood environment and family violence, it is subject to some notable limitations. The study design cannot demonstrate a causal relationship between neighborhood vacant and cited property and family violence, and there may be unidentified confounders that contribute to the associations identified. Further investigation is needed to understand if and how additional factors at the neighborhood level relate to rates of family violence. Exploration of the potential influences of neighborhood incarceration rates and over policing,^32,33^ and the presence of certain structures in the community, such as the density of liquor stores,^34^ and/or the absence of structures in the built environment (e.g., supportive community institutions) may all play a role. Additionally, given that neighbors may respond differently to safety issues such as family violence,^14,15^ research that tests tailored measures for social control and cohesion that may be more relevant to family violence is needed. Further, it is unknown whether the results are generalizable to a broader population, due to a small sample size and sociodemographic characteristics that may be specific to New Orleans. Furthermore, there is the potential for selection bias given the loss to follow-up over the study, with only 64% of the sample with data collected at 12-months. However, women lost to follow-up over the study period did not differ from those who remained in terms of sociodemographic factors, neighborhood conditions or IPV at baseline. There is also a potential for social desirability bias, due to the legal and social risks, stigma, and trauma associated with reporting family violence, as well as self-report bias. Finally, the measurement of vacant and cited properties with administrative data has certain limitations, as there can be a lack of equitable enforcement of blight violations and citations and the range of code violations that may be included in enforcement data given the range of conditions that may be cited by a particular municipality. Alternative measures of vacant and cited properties could be explored in future research.^35^

## Conclusion

Family violence occurs in the context of deeply rooted structural and social inequities that are reflected in the built environment. The findings from this study contribute to the research literature demonstrating a connection between the built environment, specifically neighborhood vacant and cited property, and family violence, including child maltreatment and IPV. Traditionally, many family violence prevention and intervention efforts have focused on interventions at the individual- and relational-level. Consequently, our findings support those from previous studies^10,26^ that neighborhood environment interventions might be an avenue for family violence prevention. Community-engaged efforts to improve the built environment by mitigating or maintaining neighborhood vacant and cited property may continue to be a focus of interventions for reducing family violence; however, further research is needed to assess the impact of various elements of disordered neighborhoods on family violence outcomes and to understand mediating and moderating effects to better refine prevention strategies and optimize outcomes. Further, due to high housing costs, the negative effects of gentrification, and the association between concentrated disadvantage and neighborhood disorder, it is also important to consider potential mitigation measures for unintended consequences such as displacement.

## Figures and Tables

**Table 1.** Characteristics of Mothers at Baseline, Overall and by Neighborhood Vacant and Cited Property Rate (N=215)

Characteristics	Overall N	Overall %	High Vacant and Cited Property Rate (n=54)		Low Vacant and Cited Property Rate (n=161)		p-value^a^
			N	%	N	%	

**Maternal Age**							.001
<25	84	39.25	31	58.49	53	32.92	
25-30	54	25.23	14	26.42	40	24.84	
31-35	47	21.96	5	9.43	42	26.09	
>=35	29	13.55	3	5.66	26	16.15	
**Maternal Education**							.000
Less than High School	49	22.79	19	35.19	30	18.63	
High School Graduate or GED	36	16.74	17	31.48	19	11.80	
Vocational / Technical / Associate Degree	17	7.91	6	11.11	11	6.83	
Some College, No Degree	41	19.07	10	18.52	31	19.25	
Bachelor’s Degree or higher	72	33.49	2	3.70	70	43.48	
**Maternal Self-Reported Race**							.002
Black	139	64.65	45	83.33	94	58.39	
White	58	26.98	5	9.26	53	32.92	
Other Asian	18	8.37	4	22.22	14	77.78	
**Household Annual Income**							.032
24,999 or less	101	50	31	63.27	70	45.75	
> $25,000	101	50	18	36.73	83	54.25	
**Maternal Relationship Status**							.000
Married or in committed relationship	113	52.56	16	29.63	97	60.25	
Single, never married or divorced	102	47.44	38	70.37	64	39.75	
**Adverse Childhood Experiences**							.162
≥4	39	18.31	13	25.00	26	16.15	
< 4	174	81.69	39	75.00	135	83.85	
**Child Maltreatment (yes)**	50	45.87	14	60.87	36	41.86	.104
**Intimate Partner Violence (yes)**	135	62.79	29	53.70	106	65.84	.113
		**Mean**		**Mean**		**Mean**	
**Average Perceived Stress Score** **(range 0-12)**	--	--	53	5.08	160	4.64	.345
**Average Rini Score (range 10-40)**	--	--	49	16.78	148	17.33	.504

Note. Based on non-missing values; less than 10% missing for each variable.

a Likelihood Ratio Chi-square or Fisher’s Exact or t-test.

**Table 2.** Association Between Neighborhood Vacant and Cited Property Rate and Family Violence: Results of Crude and Adjusted Logistic Models

	Crude Models		Adjusted Models^a^	
	Odds Ratio	95%CI	Odds Ratio	95% CI
**Child Maltreatment**				
Model 1: High vacant and cited property rate (Dichotomous)	2.11	(1.03, 4.31)	9.20	(1.03, 82.44)
Model 2: Vacant and cited property rate (Continuous)	1.01	(1.00, 1.03)	1.05	(1.00, 1.10)
**Intimate Partner Violence**				
Model 3: High vacant and cited property rate (Dichotomous)	2.52	(1.21, 5.25)	1.85	(0.88, 3.88)
Model 4: Vacant and cited property rate (Continuous)	1.02	(1.01, 1.04)	1.01	(1.00, 1.02)

a Adjusted for maternal age, education, race, relationship status, ACE history, perceived stress, and anxiety.
